# Lung Cancer in the French West Indies: Role of Sugarcane Work and Other Occupational Exposures

**DOI:** 10.3390/ijerph192013444

**Published:** 2022-10-18

**Authors:** Léïla Cabréra, Aviane Auguste, Léah Michineau, Clarisse Joachim, Jacqueline Deloumeaux, Danièle Luce

**Affiliations:** 1 Irset (Institut de recherche en santé, environnement et travail)-UMR_S 1085, EHESP, Inserm, Univ Rennes, F-97100 Pointe-à-Pitre, Guadeloupe, France; 2 Martinique Cancer Registry, UF 1441 Registre des cancers, Pôle de Cancérologie Hématologie Urologie Pathologie, University Hospital of Martinique, F-97200 Fort-de-France, Martinique, France; 3 General Cancer Registry of Guadeloupe, University Hospital of Guadeloupe, F-97100 Pointe-à-Pitre, Guadeloupe, France

**Keywords:** lung cancer, farm workers, occupational exposure, Caribbean, pesticides, herbicides

## Abstract

Objective: Our aim was to study the role of occupational exposures in lung cancer risk in the French West Indies, with special attention to some specific activities, such as sugarcane work, that can only be studied in a limited number of populations. Methods: We used data from a population-based case-control study that included 147 incident lung cancer cases and 405 controls. Smoking histories and detailed occupational histories with descriptions of tasks and substances were collected by questionnaire during face-to-face interviews. Odds ratios (OR) adjusted for sex, age, region, smoking status, and cigarette pack-years and 95% confidence intervals (95% CI) were estimated by unconditional logistic regression. Results: Significantly increased risks of lung cancer were found in sugarcane farm workers (OR = 2.7; 95% CI 1.1–6.6) and more generally in the sugarcane-growing sector (OR = 2.5; 95% CI 1.0–6.3) and to a lesser extent in rum production. Elevated risks of lung cancer were also observed among other agricultural workers, painters, warehouse porters, labourers, and maintenance and motor vehicle repair workers. Exposure to herbicides in sugarcane cultivation was associated with an increased risk of lung cancer (OR = 2.6; 95% CI 0.9–7.6). Conclusion: These results show that occupational exposures contributed to lung cancer risk in the French West Indies, and highlighted the role of exposures related to sugarcane work.

## 1. Introduction

Lung cancer is the leading cancer in the world in terms of incidence and mortality. In 2018, it is estimated that there were nearly 2.1 million new cases of lung cancer and 1.8 million deaths [1].

The main risk factor for lung cancer is smoking, which is responsible for at least 8 out of 10 lung cancer cases in Western countries. The risk of lung cancer development is around 10 times higher in smokers than in non-smokers, and the risk increases with the duration of smoking and the amount of cigarettes smoked for all histological types of lung cancer [2]. Environmental exposure to radon is considered the second most important cause of lung cancer, after smoking [3].

Besides smoking and radon, many other risk factors for lung cancer have been identified. A large proportion of these are substances or exposure situations present mainly in the workplace, and lung cancer is the most common occupational cancer. Several occupational exposures are known risk factors for lung cancer, including asbestos, crystalline silica, diesel exhaust, polycyclic aromatic hydrocarbons (PAHs), various metals (arsenic, cadmium, beryllium, some chromium and nickel compounds), welding fumes, and ionising radiation. There is remaining uncertainty with regard to several probable occupational lung carcinogens, such as bitumen or non-arsenical insecticides [4].

Guadeloupe and Martinique, known as the French West Indies (FWI), are French overseas territories in the Caribbean with a population of around 800,000, mostly Afro-Caribbean. The age-standardised (world) lung cancer incidence rates per 100,000 over the period 2007–2016 are 12.1 in Guadeloupe and 10.3 in Martinique for men; for women, these rates are 4.4 and 6.3, respectively [5].

This relatively low incidence is mainly due to low tobacco consumption [6]. Indeed, the proportion of current smokers in the FWI is 23% among men and 13% among women. The proportion of those who have ever smoked tobacco is 38% among men and 19% among women. This population with a low prevalence of smoking is therefore of particular interest for the study of other risk factors for lung cancer, foremost among which are occupational exposures. The involvement of risk factors other than tobacco is also suggested by a descriptive study on lung cancers in Guadeloupe [7], which showed a high proportion of non-smoking cases.

The FWI population presents particularities in terms of occupational risks, with specific activities such as banana cultivation and sugarcane cultivation. It has been suggested that there is excess lung cancer among banana plantation workers [8]. Elevated risks of lung cancer have also been found in sugarcane cultivation or the sugarcane industry [9]. However, the number of studies is limited, and these results need to be replicated in other appropriate populations. As in other tropical regions, the FWI are also characterised by a significant use of pesticides. Apart from these specific exposures, the high proportion of very small enterprises (more than 90%) and the high frequency of informal employment are associated with poor working conditions.

The role of occupational exposures in the aetiology of lung cancer in the Caribbean remains largely unknown. Therefore, the objective was to examine the associations between occupational exposures and lung cancer risk in the FWI and to clarify the impact of certain tasks and substances on lung cancer risk.

## 2. Methods

This work is based on data from a population-based case-control study conducted in Guadeloupe and Martinique between 2013 and 2016. This study represents an extension to the FWI of the ICARE study, a large case-control study on respiratory cancers conducted in mainland France [10], and used the same protocol and questionnaire, with some adaptations to the local context. The study included both lung cancer and head and neck cancer cases, with a common control group. The present analysis included only lung cancer cases and controls.

The cases were identified in collaboration with the cancer registries of the two regions. These registries have been in place since 1983 for Martinique and 2008 for Guadeloupe, and they record all cancer cases in each of these regions. Eligible cases were all patients with a newly diagnosed primary tumour of the trachea, bronchus, and lung (International Classification of Diseases, 10th revision, codes C33–C34) diagnosed between 1 April 2013 and 31 December 2016, residing in Guadeloupe or Martinique, and aged 75 years or younger at diagnosis. All histological types were included.

The control group was a random sample of the general population recruited by random digit dialling, using incidence density sampling method. Controls were frequency matched to all the cases by sex, age, and region. Additional stratification was used to obtain a distribution by socio-professional category comparable to that of the population (obtained from census data), to control for potential selection bias resulting from differential participation rates by socio-professional category. The procedure is described in details elsewhere [10], the only difference being that in the FWI, cell phones were also included.

Of the 237 cases identified as potentially eligible for the study, 169 (71.3%) agreed to participate and were interviewed. Of these, after review of the diagnosis, 22 (13.0%) did not meet the inclusion criteria. Of the 497 eligible controls, 405 (81.5%) completed the questionnaire. A total of 147 incident lung cancer cases and 405 controls were included in the study, for a total of 552 subjects. Each subject included in the study gave written informed consent. Cases and controls were interviewed by specially trained interviewers, with a standardised questionnaire including socio-demographic characteristics; smoking habits; detailed occupational history, with a description of each job held (work position, company activity, tasks performed); and specific questionnaires for certain occupations or activities. These job-specific questionnaires included a comprehensive list of questions on exposures, tasks performed, and materials handled. In addition to the job-specific questionnaires originally used in the ICARE study [10], we developed a questionnaire for the sugarcane industry, and we modified the agriculture questionnaire to better describe local agricultural activities.

For each subject, all jobs performed during his or her working life (occupation and industry) were coded, using the International Standard Classification of Occupations (ISCO) of the International Labour Office of 1968 for the occupation and the Nomenclature d’Activités Française (NAF) of the INSEE of 2000 for the industry. The coding was performed by a trained coder blind to case-control status. We then generated dichotomous variables for each occupation/industry: “having ever worked in a given occupation/industry” versus “having never worked in this occupation/industry” using three ISCO levels (1, 2, and 5-digit codes) and two NAF levels (2- and 4-digit codes). We were also interested in tasks and substances in the workplace known or suspected to be associated with an increased risk of lung cancer. Algorithms were created from the responses to the different questionnaires in order to assess exposure to asbestos, diesel or gasoline exhaust, fumes, dusts, solvents, or welding. The agriculture and sugarcane industry questionnaires were analysed in detail, to evaluate exposure to pesticides and several tasks related to sugarcane work. All exposures were evaluated in two categories: ever exposed vs. never exposed.

Unconditional logistic regression models were used to estimate adjusted odds ratios (ORs) and their 95% confidence intervals (95% CIs) and to assess interactions. ORs were adjusted for sex, age (continuous), region (Guadeloupe or Martinique), smoking status (never smoker, ex-smoker, current smoker), and cumulative cigarette quantity in pack-years (continuous). Never smokers are those who have smoked fewer than 100 cigarettes in their lifetime. Ex-smokers are those who quit smoking at least two years previously.

## 3. Results

Our study included 156 women (57 cases, 99 controls) and 396 men (90 cases, 306 controls). As expected, the proportion of ever smokers was higher in the cases than in the controls (59% vs. 34% respectively), and the risk of lung cancer was significantly increased in ex-smokers (OR = 2.5; 95% CI 1.4–4.3) and in current smokers (OR = 8.5; 95% CI 4.8–14.8). The risk also increased with the cumulative cigarette quantity. The proportion of those with primary education or less was higher among cases than among controls, but educational level was not significantly associated with lung cancer risk. Examination of the distribution of cases according to histological type showed a high proportion of adenocarcinomas (62%), followed by squamous cell carcinomas (19%) and other histological types (19%). The main characteristics of the study population are presented in Supplementary Materials Table S1.

Table 1 presents the data related to the study of occupations of the subjects. For the three ISCO code levels, ORs are presented only for occupations with at least five exposed cases. Overall, the ORs for professional, technical and related workers, clerical and related workers, and sale workers (ISCO codes 0/1 to 4) were less than 1. However, a slight increase in risk was observed for service workers (ISCO code 5); agricultural, animal husbandry, and forestry workers, fishermen, and hunters (ISCO code 6); and production and related workers, transport equipment operators, and labourers (ISCO code 7/8/9).

Looking in more detail at the next ISCO level, 2-digit codes, borderline significant ORs greater than 1 were observed for agricultural and animal husbandry workers (ISCO code 62) and labourers not elsewhere classified (ISCO code 99), 1.5 and 2.3, respectively. High, although not significant, ORs were also observed for painters (ISCO code 93), bricklayers, carpenters, and other construction workers (ISCO code 95) and material handling and related equipment operators, dockers, and freight handlers (ISCO code 97).

A more detailed analysis for agricultural and animal husbandry workers showed a significantly increased risk (OR = 2.7; 95% CI 1.1–6.6) for sugarcane farm workers (ISCO code 62260). There was some indication of an increase in risk with the duration of employment (≤15 years: OR = 2.2, 95% CI 0.8–6.4; >15 years: OR = 4.4, 95% CI 0.9–23.1). We also observed a moderate increase in risk for gardeners (ISCO code 62740) and field crop farm workers (ISCO code 62210). It should be noted that this code includes banana workers, who cannot be clearly identified by ISCO. The increase in risk observed for the category of “material handling and related equipment operators, dockers and freight handlers” only concerned warehouse porters (ISCO code 97145). The labourers subgroup included only one occupation. For bricklayers, carpenters, and other construction workers, no high OR was observed at the 5-digit ISCO level with at least five exposed cases.

Table S2 in Supplementary Materials shows the ORs associated with occupations in men and women. This analysis did not reveal any specific findings, with the exception of a non-significantly elevated risk of lung cancer among male cooks, waiters, and bartenders (OR = 3.0; 95% CI 0.8–11.4). The number of women was too small in most occupations for a meaningful analysis. Occupations with excess risks were predominantly male occupations.

ORs associated with the different industries (2- and 4-character NAF codes) are presented in Table 2, for industries with at least five exposed cases. A non-significant increase in lung cancer risk was observed in agriculture, hunting, and related service activities (NAF code 01). This increase was limited to the growing of cereals and other crops n.e.c. and the growing of fruit. The growing of cereals and other crops n.e.c (NAF code 011A) corresponded here exclusively to sugarcane cultivation, for which a significant OR of 2.5 was observed (OR = 2.5; 95% CI 1.0–6.3), consistent with the high OR found for sugarcane farm workers, but with no trend with employment duration (≤15 years: OR = 2.6, 95% CI 0.8–8.6; >15 years: OR = 2.3, 95% CI 0.6–9.1). The growing of fruit (NAF code 011F) corresponded to banana cultivation, associated with a non-significant OR of 1.1. The OR of 1.7 associated with the manufacture of distilled potable alcoholic beverages (NAF code 159A, here, rum manufacturing) was consistent with the risk increases observed for other sugarcane workers. This increase in risk among rum-manufacturing workers was limited to those who had worked more than 15 years (≤15 years: OR = 1.0, 95% CI 0.2–4.2; >15 years: OR = 4.5, 95% CI 0.8–26.4). High but non-significant ORs were also observed in the maintenance and repair of motor vehicles (NAF code 502Z), the wholesale and retail trade (NAF codes 51 and 52, respectively), general (overall) public service activities (NAF code 751A), primary education (NAF code 801Z) as well as technical and vocational secondary education (NAF code 802C), and finally other service activities (NAF code 93) and activities of households as employers of domestic staff (NAF code 950Z).

In addition to this analysis of job titles, a number of exposure situations known or suspected to be associated with an increased risk of lung cancer were further analysed (Table 3). Although not significant, high ORs were found for exposures to diesel and gasoline exhaust, paint, fumes, solvents, wood treatment products, and disinfection of agricultural premises. Of note, the OR for exposure to fumes was borderline significant (OR = 1.4; 95% CI 0.9–2.3). Conversely, no association was found for exposure to dusts, acids, welding, or asbestos. In our study, exposure to asbestos was related primarily to work with asbestos-containing materials (insulation materials, flocked surfaces, or asbestos cement), the use of asbestos gaskets or filters, or brake maintenance and repair. None of these tasks were associated with an increased risk, but the analysis was hampered by small numbers (Table S3 in Supplementary Materials).

We analysed occupational exposure to pesticides (i.e., herbicides, insecticides, fungicides, and other biocides) in sugarcane cultivation, banana cultivation, and other crops. A strong, borderline significant increase in risk was associated with sugarcane pesticides (OR = 2.6; 95% CI 0.9–7.6). This increase was entirely due to herbicides, as none of the subjects in our study reported applying either insecticides or fungicides to sugarcane. An increased risk (OR = 3.0; 95% CI 0.8–10.5) was also found for banana pesticides. Again, this increase was mainly due to the application of herbicides (OR = 3.2; 95% CI 0.9–11.6). Insecticides and herbicides used on other crops were associated with lower and non-significant ORs. No association was found with the use of fungicides.

We examined certain tasks related to sugarcane cultivation in greater detail (Table 3). The activities of cutting, collecting, and crushing sugarcane, as well as handling, were associated with a non-significant increase in lung cancer risk, with respective ORs of 1.4, 1.8, 3.7, and 1.9. No association was found with the burning of sugarcane before or after harvesting.

For 24 cases who were too ill to answer the full questionnaire, a shorter version of the questionnaire was used, which included fewer details on tasks and exposures. We conducted a sensitivity analysis after excluding these subjects (Table S4 in Supplementary Materials). The results were not markedly changed, but the ORs were in general slightly increased and reached statistical significance for herbicides in sugarcane (OR = 3.2; 95% CI 1.1–9.3) and banana (OR = 3.4; 95% CI 1.0–11.8) cultivation and for sugarcane handling (OR = 2.5; 95% CI 1.0–6.3).

## 4. Discussion

To our knowledge, this is the first study on the occupational risk factors for lung cancer in the Caribbean. As is classically observed in lung cancer studies, the proportion of smokers was higher in cases than in controls; however, in our study, the proportion of cases who had never smoked amounted to 40% and was higher than the values usually observed, which are typically in the order of 10 to 20%. This means that in these regions, other factors are indeed involved in the occurrence of lung cancer. Examination of the distribution of cases according to histological type showed a high proportion of adenocarcinomas (62%), which was consistent with the predominance of adenocarcinoma among non-smoking cases previously reported in the literature [11,12].

Analyses according to occupation and industry showed few significant results, but the elevated, although not significant, ORs observed for painters, warehouse porters, labourers, and motor vehicle repairers are consistent with the literature. Occupational exposure as a painter has been classified as a lung carcinogen by the International Agency on Research for Cancer [13], and a recent pooled analysis of 16 case-control studies reported an OR of 1.3 associated with ever having worked as a painter [14]. An elevated risk of lung cancer among dockers and freight handlers was found in several studies [15,16,17]. Several studies also reported a slight increase in lung cancer risk among workers engaged in motor vehicle repair [18].

Similarly, results for exposures and tasks were mainly nonsignificant, but the non-significantly increased OR observed for diesel exhaust is in agreement with existing knowledge [19,20]. On the other hand, some known associations were not found. In particular, we did not observe an increase in risk among those who had performed welding activities, contrary to other studies [21]. Nor did we observe any association with exposure to asbestos, a well-known lung carcinogen. These results may be explained by low exposure levels. In our study, welding activities were mainly sporadic tasks, resulting in an overall low level of exposure. The same is true for asbestos exposure. Exposure levels were probably low even if they were not formally assessed. The occasional tasks that resulted in asbestos exposure in our study are unlikely to have generated very high levels of exposure. Activities entailing high exposure levels such as the mining and milling of asbestos or the manufacture of asbestos-containing products were never present in the FWI. Our findings are also likely to be affected by non-differential misclassification of exposure (see below), which biases the estimates towards the null [22]. Some other studies found no association between lung cancer and asbestos exposure [23,24], and non-differential misclassification combined with low exposure levels was suggested as plausible explanations.

The most striking finding of our study is the significantly increased risk of lung cancer in sugarcane farm workers and in sugarcane growing in general, as well as a non-significant increase in risk in rum manufacturing. Although the number of studies is limited, excess lung cancer risk has been observed in sugarcane growing or the sugarcane industry [9,25,26]. This excess could be due to the presence of biogenic amorphous silica fibres in sugarcane leaves, which have characteristics similar to those of asbestos [27]. We found elevated ORs associated with cutting, crushing, collecting, or handling the cane. These fibres can also be transformed into crystalline silica at high temperatures, during sugar or rum production, or during the burning of cane before or after cutting. Another exposure that may arise from sugarcane burning is exposure to polycyclic aromatic hydrocarbons (PAHs), a known risk factor for lung cancer [28,29]. However, sugarcane burning was not associated with lung cancer risk in our study.

In contrast, the use of herbicide treatments in sugarcane was associated with an increased risk of lung cancer. Herbicide exposure in banana cultivation and to a lesser extent in other crops was also associated with an elevated risk of lung cancer. It was not possible to analyse chemical families or active substances, as the products used were not always clearly identified in the questionnaires but rather identified by trade names, and not all exposed subjects could recall them. The most frequently cited herbicides are glyphosate, paraquat, phenoxy herbicides, and triazines. The use of arsenical herbicides in sugarcane cultivation has been reported in the United States, notably in Hawaii [30]. To our knowledge, they have not been used in the FWI, and none of the subjects in our study reported exposure to arsenic or arsenical pesticides. Few studies have been conducted to assess the relationship between lung cancer and specific substances used as pesticides. The results come almost exclusively from the Agricultural Health Study [31] and do not show an association between lung cancer risk and the herbicides mentioned by the participants in our study. Conversely, associations between lung cancer risk and several insecticides used in the FWI have been suggested, including carbamates, organophosphates such as diazinon, and organochlorines such as dieldrin and lindane [31,32]. No clear association with insecticide exposure, however, was found in our study.

A limitation of our study is the relatively small number of cases and the consequently limited statistical power. Misclassification of exposure may also have occurred, which is likely to be non-differential. Self-reported occupational history is generally considered reliable [33]. Coding was performed by a unique trained coder, blind to case-control status; therefore, if coding errors were made, they were non-differential. Exposures were assessed from self-reported information on tasks performed and materials handled. The assessment was performed automatically regardless of case-control status, also resulting in non-differential misclassification of exposure. For dichotomized exposures, non-differential exposure misclassification tends to bias the OR towards the null and is therefore unlikely to explain our positive findings. On the other hand, some tasks may have not been accurately reported, and recall bias, i.e., the fact that reporting differs between cases and controls, may be a concern. Investigators who were able to compare the accuracy of occupational exposure reporting between cases and controls found little or no difference [33]. Our sensitivity analysis, after excluding those who answered a shorter version of the questionnaire, presumably with lower-quality data, resulted in increased ORs. Altogether, we believe that the ORs in our study are more likely to be underestimated than overestimated. Finally, data on the duration and frequency of exposure to certain tasks and substances were sometimes missing, and we were not able to assess exposure levels. This relatively crude exposure assessment combined with the small sample size precluded in-depth analyses by subgroups and the study of dose–response relationships.

Nevertheless, our study has several strengths. Participation rates were satisfactory for a population-based study. Given the involvement of local cancer registries in case selection and the methods used to select controls, we believe that no major selection bias occurred in this study. Cases included in the study had a distribution by sex, age, and histological type similar to that of all the cases recorded by the registries; therefore, our cases can be considered representative of the lung cancer cases in the FWI. The method used to select our control group was previously demonstrated to yield unbiased samples. The control group was a random sample, with a distribution by socio-professional category similar by design to that of the general population. The distribution of educational level and smoking status in our control group was also close to that observed in a health survey conducted in Guadeloupe and Martinique in 2014 in a representative sample of the population [6]. Our controls can therefore reasonably be considered representative of persons with similar sex and age from the general population. Although residual confounding cannot be excluded, we carefully adjusted for smoking, the most important risk factor for lung cancer. We also used detailed questionnaires allowing us to identify a substantial amount of information on the subjects’ occupational history, aside from job titles alone. Finally, our study was able to examine occupational exposures in activities that can only be studied in a limited number of populations, such as sugarcane work or banana cultivation.

## 5. Conclusions

Our study showed that occupational risk factors contributed to the occurrence of lung cancer in the FWI and confirmed the role of specific exposures related to sugarcane work in lung cancer risk. Our findings highlighted the role of herbicides used in sugarcane cultivation and suggest more generally an association between exposure to herbicides and lung cancer.

## 6. Strengths and Limitations of This Study

- This population-based study was conducted in an Afro-Caribbean population, with specific and rarely studied occupational exposures, such as those related to banana or sugarcane cultivation.
- Detailed information on lifetime occupational history was obtained by in-person interviews.
- Misclassification of exposure may have occurred, which is likely to be non-differential.
- A limitation, common in studies of small populations, is the relatively small number of cases.

## Figures and Tables

**Table 1:** Association between selected occupations and lung cancer risk in the French West Indies (FWI).

Occupations #	ISCO Codes	Cases *n* = 147	Controls *n* = 405	OR *	95% CI
Professional, technical, and related workers	0/1	40	121	0.8	0.5–1.3
Clerical and related workers	3	34	119	0.7	0.4–1.1
Sale workers	4	24	72	0.8	0.4–1.4
Service workers	5	42	106	1.1	0.6–1.8
Cooks, waiters, bartenders, and related Workers	53	8	28	0.9	0.3–2.1
Maids and related housekeeping service workers not elsewhere classified	54	15	36	0.9	0.4–1.8
Building caretakers, charworkers, cleaners, and related workers	55	8	27	0.9	0.4–2.1
Service workers not elsewhere classified	59	7	13	1.0	0.4–2.9
Agricultural, animal husbandry, and forestry workers, fishermen, and hunters	6	29	78	1.2	0.7–2.1
Agricultural and animal husbandry workers	62	26	54	1.5	0.8–2.8
Field crop farm worker (general)	62210	6	8	1.5	0.5–4.8
Sugarcane farm worker	62260	13	18	2.7	1.1–6.6
Gardener	62740	7	14	1.8	0.6–5.5
Production and related workers, transport equipment operators, and labourers	7/8/9	58	192	1.1	0.7–1.8
Painters	93	7	13	1.3	0.4–3.7
Bricklayers, carpenters, and other construction workers	95	18	58	1.1	0.5–2.1
Reinforced concreter (general)	95210	9	36	0.9	0.4–2.2
Material handling and related equipment operators, dockers, and freight handlers	97	13	30	1.8	0.8–4.0
Warehouse porter	97145	6	11	2.4	0.8–7.3
Transport equipment operators	98	14	47	1.0	0.5–2.2
Labourers	99910	10	22	2.3	0.9–5.9

# Occupations with at least 5 cases. * ORs adjusted for sex, age (continuous), region, smoking status (never smokers, ex-smokers, smokers), and cumulative quantity of cigarettes in pack-years (continuous).

**Table 2:** Association between selected industries and lung cancer risk in the French West Indies.

Industries #	NAF Codes	Cases *n* = 147	Controls *n* = 405	OR *	95% CI
Agriculture, hunting, and related service activities	01	21	54	1.4	0.7–2.6
Growing of cereals and other crops n.e.c.	011A	12	19	2.5	1.0–6.3
Growing of fruit, nuts, beverage, and spice crops	011F	7	16	1.1	0.4–2.9
Manufacture of food products, beverages, and tobacco	15	13	44	0.9	0.4–1.9
Manufacture of distilled potable alcoholic beverages	159A	8	11	1.7	0.6–5.3
Construction	45	22	100	0.7	0.4–1.3
Construction of single-family houses	452A	8	35	0.6	0.2–1.6
General construction of building and civil engineering works	452B	6	28	0.8	0.3–2.3
Sale, maintenance, and repair of motor vehicles and motorcycles; retail sale of automotive fuel	50	10	33	1.1	0.5–2.5
Maintenance and repair of motor vehicles	502Z	8	13	2.2	0.8–5.9
Wholesale trade and commission trade, except of motor vehicles and motorcycles	51	10	20	1.3	0.5–3.1
Retail trade, except of motor vehicles and motorcycles and personal and household goods	52	33	67	1.5	0.8–2.5
Retail sale of food and beverages in non-specialized stores	521B	8	14	1.4	0.5–4.0
Hotels and restaurants	55	10	32	0.9	0.4–2.0
Land transport; transport via pipelines	60	6	33	0.4	0.1–1.1
Other business activities	74	6	37	0.4	0.2–1.2
Public administration and defence; compulsory social security	75	36	147	0.6	0.3–0.9
General (overall) public service activities	751A	21	49	1.2	0.7–2.2
Defence activities	752C	12	101	0.2	0.1–0.5
Education	80	30	109	0.9	0.5–1.5
Primary education	801Z	10	23	1.3	0.6–3.1
General secondary education	802A	8	30	0.8	0.3–2.1
Technical and vocational secondary education	802C	8	23	2.1	0.8–5.3
Adult and other education n.e.c.	804C	5	31	0.4	0.2–1.3
Health and social work	85	20	53	0.7	0.4–1.4
Hospital activities	851A	12	28	0.9	0.4–2.0
Activities of membership organisations n.e.c.	91	5	9	0.7	0.2–2.0
Recreational, cultural, and sporting activities	92	5	25	0.7	0.2–1.9
Other service activities	93	5	7	1.6	0.3–7.4
Activities of households as employers of domestic staff	95	17	37	1.1	0.5–2.1

# Industries with at least 5 cases. * ORs adjusted for sex, age (continuous), region, smoking status (never smokers, ex-smokers, smokers), and cumulative quantity of cigarettes in pack-years (continuous).

**Table 3:** Association between occupational exposures and specific tasks and lung cancer risk in the French West Indies.

Exposures	Cases *n* = 147	Controls *n* = 405	OR *	95% CI
Diesel exhaust	22	54	1.4	0.7–2.6
Gasoline exhaust	18	48	1.2	0.6–2.4
Dusts	66	235	0.8	0.5–1.2
Fumes	43	99	1.4	0.9–2.3
Acids	25	87	0.8	0.4–1.4
Welding	9	49	0.7	0.3–1.6
Painting	21	54	1.3	0.7–2.4
Asbestos	13	75	0.6	0.3–1.2
Solvents	24	60	1.3	0.7–2.4
Wood treatment products	9	15	1.5	0.5–4.1
Other chemical products	4	5	1.4	0.2–7.5
Disinfection of agricultural premises	4	11	1.6	0.4–7.0
Pesticides ^a^ in general ^b^	13	36	1.6	0.7–3.5
Pesticides in sugarcane	8	13	2.6	0.9–7.6
Pesticides in banana	6	8	3.0	0.8–10.5
Pesticides in other crops	4	21	1.2	0.4–3.8
Insecticides in general	4	17	1.2	0.4–4.0
Insecticides in sugarcane	0	0	-	-
Insecticides in banana	1	6	0.7	0.1–6.9
Insecticides in other crops	3	10	1.7	0.4–7.1
Herbicides in general	13	32	1.9	0.8–4.1
Herbicides in sugarcane	8	13	2.6	0.9–7.6
Herbicides in banana	6	7	3.2	0.9–11.6
Herbicides in other crops	4	16	1.7	0.5–5.6
Fungicides in general	2	18	0.8	0.2–3.6
Fungicides in sugarcane	0	0	-	-
Fungicides in banana	1	5	1.8	0.2–16.7
Fungicides in other crops	1	12	0.6	0.1–4.8
Sugarcane work				
Cutting of the sugarcane	9	18	1.4	0.5–3.8
Collecting the sugarcane	6	16	1.8	0.6–5.4
Burning of the sugarcane	4	15	0.6	0.1–2.1
Other work in the field	4	14	0.9	0.2–3.6
Crushing of the sugarcane	2	1	3.7	0.1–125.3
Handling the sugarcane	10	20	1.9	0.7–4.7

* ORs adjusted for sex, age (continuous), region, smoking status (never smokers, ex-smokers, smokers), and cumulative quantity of cigarettes in pack-years (continuous). ^a^ Pesticides include insecticides, herbicides and fungicides; ^b^ “in general” stands for all crops combined.

## Data Availability

The data that support the findings of this study are available from the corresponding author, upon reasonable request.

## Supplementary Materials

The following supporting information can be downloaded at: https://www.mdpi.com/article/10.3390/ijerph192013444/s1, Table S1: Main characteristics of cases and controls., Table S2. Association between selected occupations and lung cancer risk in the French West Indies among men and women., Table S3. Association between asbestos-related tasks and lung cancer risk in the French West Indies, Table S4. Association between occupational exposures and specific tasks and lung cancer risk in the French West Indies. Analysis restricted to full questionnaires.

## References

[1] Ferlay J., Colombet M., Soerjomataram I., Mathers C., Parkin D.M., Piñeros M., Znaor A., Bray F. (2019). Estimating the Global Cancer Incidence and Mortality in 2018: GLOBOCAN Sources and Methods. Int. J. Cancer.

[2] IARC (2004). Tobacco Smoke and Involuntary Smoking. IARC Monographs on the Evaluation of Carcinogenic Risks to Humans.

[3] Gaskin J., Coyle D., Whyte J., Krewksi D. (2018). Global Estimate of Lung Cancer Mortality Attributable to Residential Radon. Environ. Health Perspect..

[4] Delva F., Andujar P., Lacourt A., Brochard P., Pairon J.-C. (2016). [Occupational risk factors for lung cancer]. Rev. Mal. Respir..

[5] Estimations régionales et départementales de l’incidence et de la mortalité par cancer en France, 2007–2016. https://www.santepubliquefrance.fr/maladies-et-traumatismes/cancers/estimations-regionales-et-departementales-de-l-incidence-et-de-la-mortalite-par-cancer-en-france-2007-2016.

[6] Auguste A., Dugas J., Menvielle G., Barul C., Richard J.-B., Luce D. (2019). Social Distribution of Tobacco Smoking, Alcohol Drinking and Obesity in the French West Indies. BMC Public Health.

[7] Cadelis G., Kaddah S., Bhakkan B., Quellery M., Deloumeaux J. (2013). [Epidemiology and incidence of primary lung cancer in a region with low tobacco consumption: Guadeloupe (French West Indies). Data from the cancer registry 2008–2009]. Rev. Mal. Respir..

[8] Hofmann J., Guardado J., Keifer M., Wesseling C. (2006). Mortality among a Cohort of Banana Plantation Workers in Costa Rica. Int. J. Occup. Environ. Health.

[9] Amre D.K., Infante-Rivard C., Dufresne A., Durgawale P.M., Ernst P. (1999). Case-Control Study of Lung Cancer among Sugar Cane Farmers in India. Occup. Environ. Med..

[10] Luce D., Stücker I. (2011). ICARE Study Group Investigation of Occupational and Environmental Causes of Respiratory Cancers (ICARE): A Multicenter, Population-Based Case-Control Study in France. BMC Public Health.

[11] Subramanian J., Govindan R. (2007). Lung Cancer in Never Smokers: A Review. J. Clin. Oncol..

[12] Siegel D.A., Fedewa S.A., Henley S.J., Pollack L.A., Jemal A. (2021). Proportion of Never Smokers Among Men and Women With Lung Cancer in 7 US States. JAMA Oncol..

[13] IARC Working Group on the Evaluation of Carcinogenic Risks to Humans (2012). Chemical Agents and Related Occupations. IARC Monographs on the Evaluation of Carcinogenic Risks to Humans.

[14] Guha N., Bouaoun L., Kromhout H., Vermeulen R., Brüning T., Behrens T., Peters S., Luzon V., Siemiatycki J., Xu M. (2021). Lung Cancer Risk in Painters: Results from the SYNERGY Pooled Case-Control Study Consortium. Occup. Environ. Med..

[15] Bardin-Mikolajczak A., Lissowska J., Zaridze D., Szeszenia-Dabrowska N., Rudnai P., Fabianova E., Mates D., Navratilova M., Bencko V., Janout V. (2007). Occupation and Risk of Lung Cancer in Central and Eastern Europe: The IARC Multi-Center Case–Control Study. Cancer Causes Control.

[16] Brüske-Hohlfeld I., Mühner M., Pohlabeln H., Ahrens W., Bolm-Audorff U., Kreienbrock L., Kreuzer M., Jahn I., Wichmann H.-E., Jückel K.-H. (2000). Occupational Lung Cancer Risk for Men in Germany: Results from a Pooled Case-Control Study. Am. J. Epidemiol..

[17] Richiardi L., Boffetta P., Simonato L., Forastiere F., Zambon P., Fortes C., Gaborieau V., Merletti F. (2004). Occupational Risk Factors for Lung Cancer in Men and Women: A Population-Based Case–Control Study in Italy. Cancer Causes Control.

[18] Goodman M., Teta M.J., Hessel P.A., Garabrant D.H., Craven V.A., Scrafford C.G., Kelsh M.A. (2004). Mesothelioma and Lung Cancer among Motor Vehicle Mechanics: A Meta-Analysis. Ann. Occup. Hyg..

[19] Olsson A.C., Gustavsson P., Kromhout H., Peters S., Vermeulen R., Brüske I., Pesch B., Siemiatycki J., Pintos J., Brüning T. (2011). Exposure to Diesel Motor Exhaust and Lung Cancer Risk in a Pooled Analysis from Case-Control Studies in Europe and Canada. Am. J. Respir. Crit. Care Med..

[20] Benbrahim-Tallaa L., Baan R.A., Grosse Y., Lauby-Secretan B., El Ghissassi F., Bouvard V., Guha N., Loomis D., Straif K. (2012). International Agency for Research on Cancer Monograph Working Group Carcinogenicity of Diesel-Engine and Gasoline-Engine Exhausts and Some Nitroarenes. Lancet Oncol..

[21] Guha N., Loomis D., Guyton K.Z., Grosse Y., El Ghissassi F., Bouvard V., Benbrahim-Tallaa L., Vilahur N., Muller K., Straif K. (2017). Carcinogenicity of Welding, Molybdenum Trioxide, and Indium Tin Oxide. Lancet Oncol..

[22] Blair A., Stewart P., Lubin J.H., Forastiere F. (2007). Methodological Issues Regarding Confounding and Exposure Misclassification in Epidemiological Studies of Occupational Exposures. Am. J. Ind. Med..

[23] Carel R., Olsson A.C., Zaridze D., Szeszenia-Dabrowska N., Rudnai P., Lissowska J., Fabianova E., Cassidy A., Mates D., Bencko V. (2007). Occupational Exposure to Asbestos and Man-Made Vitreous Fibres and Risk of Lung Cancer: A Multicentre Case-Control Study in Europe. Occup. Environ. Med..

[24] Wünsch-Filho V., Moncau J.E., Mirabelli D., Boffetta P. (1998). Occupational Risk Factors of Lung Cancer in São Paulo, Brazil. Scand. J. Work Environ. Health.

[25] Rothschild H., Mulvey J.J. (1982). An Increased Risk for Lung Cancer Mortality Associated with Sugarcane Farming. J. Natl. Cancer Inst..

[26] Brooks S.M., Stockwell H.G., Pinkham P.A., Armstrong A.W., Witter D.A. (1992). Sugarcane Exposure and the Risk of Lung Cancer and Mesothelioma. Environ. Res..

[27] Newman R.H. (1986). Fine Biogenic Silica Fibres in Sugar Cane: A Possible Hazard. Ann. Occup. Hyg..

[28] Cristale J., Silva F.S., Zocolo G.J., Marchi M.R.R. (2012). Influence of Sugarcane Burning on Indoor/Outdoor PAH Air Pollution in Brazil. Environ. Pollut..

[29] Boeniger M., Hawkins M., Marsin P., Newman R. (1988). Occupational Exposure to Silicate Fibres and PAHs during Sugar-Cane Harvesting. Ann. Occup. Hyg..

[30] Cutler W.G., Brewer R.C., El-Kadi A., Hue N.V., Niemeyer P.G., Peard J., Ray C. (2013). Bioaccessible Arsenic in Soils of Former Sugar Cane Plantations, Island of Hawaii. Sci. Total Environ..

[31] Bonner M.R., Freeman L.E.B., Hoppin J.A., Koutros S., Sandler D.P., Lynch C.F., Hines C.J., Thomas K., Blair A., Alavanja M.C.R. (2017). Occupational Exposure to Pesticides and the Incidence of Lung Cancer in the Agricultural Health Study. Environ. Health Perspect..

[32] Alavanja M.C.R. (2004). Pesticides and Lung Cancer Risk in the Agricultural Health Study Cohort. Am. J. Epidemiol..

[33] Teschke K., Olshan A.F., Daniels J.L., De Roos A.J., Parks C.G., Schulz M., Vaughan T.L. (2002). Occupational Exposure Assessment in Case-Control Studies: Opportunities for Improvement. Occup. Environ. Med..

