# Supplementary material for: Lung Cancer in the French West Indies: Role of Sugarcane Work and Other Occupational Exposures
Source: Int J Environ Res Public Health. 2022 Oct 18;19(20):13444. doi: 10.3390/ijerph192013444 (PMC9603435; doi:10.3390/ijerph192013444)
Supplement: Supplementary file 1 [file ijerph-19-13444-s001.zip › ijerph-1948120-supplementary.pdf]

Table S1. Main characteristics of cases and controls

	Cases	Controls	OR (95% CI)
	N (%)	N (%)	
**Total**	147 (100)	405 (100)	
**Sex**			
Men	90 (61,2)	306 (75,6)	
Women	57 (38,8)	99 (24,4)	
**Age (years)**			
<45	4(2.7)	62 (15.3)	
45-54	25 (17.0)	107 (26.4)	
55-64	54 (36.7)	129 (31.9)	
>65	64 (43.5)	107 (26.4)	
**Educational level***			
Up to primary education	49 (33,3)	94 (23,2)	1 (Ref)
Less than high school diploma	46 (31,3)	161 (39,8)	0,8 (0,4-1,3)
High school diploma	21 (14,3)	53 (13,1)	0,8 (0,4-1,6)
Tertiary education	31 (21,1)	97 (23,9)	0,7 (0,4-1,3)
**Smoking status** §			
Never smokers	60 (40.8)	266 (65.7)	1 (Ref)
Ex-smokers	36 (24.5)	89 (22.0)	2.7 (1.6-4.7)
Smokers	51 (34.7)	50 (12.3)	8.2 (4.7-14.5)
**Pack-years**§			
Never smokers	60 (40.8)	266 (65.7)	1 (Ref)
≤ 10	15 (10.2)	62 (15.3)	1.6 (0.8-3.2)
]10-20]	14 (9.5)	30 (7.4)	4.0 (1.9-8.6)
]20-40	29 (19.7)	30 (7.4)	7.3 (3.8-14.1)
> 40	29 (19.7)	17 (4.2)	12.8 (6.1-27.2)

* ORs adjusted for age (continuous), region, smoking status (never smokers, ex-smokers, smokers) and cumulative quantity of cigarettes in pack-years (continuous

§ ORs adjusted for sex, age (continuous), region

Table S2. Association between selected occupations and lung cancer risk in the French West Indies among men and women.

		Women				Men			
**Occupations**	**ISCO**	**Cases**	**Controls**	**OR***	**95% CI**	**Cases**	**Controls**	**OR***	**95% CI**
	codes	n=57	n=99			n=90	n=306		
Professional, technical and related workers	0/1	16	31	0.8	0.4-1.6	24	90	0.8	0.5-1.6
Clerical and related workers	3	19	32	1.0	0.5-2.1	15	87	0.5	0.2-0.9
Sale workers	4	10	18	1.1	0.4-2.8	14	54	0.6	0.3-1.3
Service workers	5	26	60	1.1	0.6-1.8	16	46	1.7	0.8-3.6
Cooks, Waiters, Bartenders and Related Workers	53	3	16	0.4	0.1-1.5	5	12	3.0	0.8-11.4
Maids and Related Housekeeping Service Workers Not Elsewhere Classified	54	14	36	0.7	0.3-1.5	1	0		
Building Caretakers, Charworkers, Cleaners and Related Workers	55	5	13	0.7	0.2-2.2	3	14	1.0	0.3-4.3
Service Workers Not Elsewhere Classified	59	4	8	0.9	0.3-3.2	3	5	1.3	0.2-7.3
Agricultural, animal husbandry and forestry workers, fishermen and hunters	6	4	5	1.2	0.3-5.5	25	73	1.3	0.7-2.4
Agricultural and Animal Husbandry Workers	62	4	5	1.2	0.3-5.5	22	49	1.7	0.9-3.4
Field crop farm worker (general)	62210	1	3			5	5	2.3	0.6-9.4
Sugar-cane farm worker	62260	1	0			12	18	3.0	1.2-7.7
Gardener	62740	1	1			6	13	2.0	0.6-6.4
Production and related workers, transport equipment operators and labourers	7/8/9	5	9	0.9	0.3-3.2	53	183	1.2	0.7-2.1
Painters	93	0	1			7	12	1.5	0.5-4.6
Bricklayers, Carpenters and Other Construction Workers	95	1	1			17	57	1.2	0.6-2.4
Reinforced concreter (general)	95210	0	0			9	36	0.9	0.4-2.2
Material Handling and Related Equipment Operators, Dockers and Freight Handlers	97	2	2			11	28	1.7	0.7-4.1
Warehouse porter	97145	0	1			6	10	3.0	0.9-9.6
Transport Equipment Operators	98	0	0			14	47	1.0	0.5-2.2
Labourers	99910	1	0			9	22	2.3	0.8-6.3

*ORs adjusted for age (continuous), region, smoking status (never smokers, ex-smokers, smokers) and cumulative quantity of cigarettes in pack-years (continuous) ; ORs were not calculated when the number of exposed cases was less than 3

Table S3. Association between asbestos-related tasks and lung cancer risk in the French West Indies.

Task	Cases	Controls	OR*	95% CI
	n=147	n=405		
Work with insulation materials	4	34	0.5	0.2-1.7
Work with asbestos cement	4	25	0.5	0.2-1.7
Work on flocked surfaces	2	11	1.0	0.2-4.7
Use of asbestos gaskets	3	16	0.8	0.2-3.2
Use of asbestos filters	1	1	5.9	0.3-117.4
Brake maintenance and repair	3	14	0.7	0.2-2.9

*ORs adjusted for age (continuous), region, smoking status (never smokers, ex-smokers, smokers) and cumulative quantity of cigarettes in pack-years (continuous)

Table S4. Association between occupational exposures and specific tasks and lung cancer risk in the French West Indies. Analysis restricted to full questionnaires.

Exposures	Cases	Controls	OR*	95% CI
	n=123	n=405		
Diesel exhaust	20	54	1.5	0.8-3.0
Gasoline exhaust	16	48	1.4	0.7-2.7
Dusts	62	235	1.0	0.6-1.6
Fumes	40	99	1.6	0.9-2.6
Acids	24	87	0.9	0.5-1.7
Welding	8	49	0.8	0.3-1.9
Painting	18	54	1.3	0.7-2.6
Asbestos	13	75	0.8	0.4-1.5
Solvents	23	60	1.6	0.8-2.9
Wood treatment products	8	15	1.6	0.5-4.5
Other chemical products	4	5	1.5	0.3-8.5
Disinfection of agricultural premises	4	11	1.9	0.4-8.0
Pesticides[a] in general[b]	13	36	1.9	0.9-4.2
Pesticides in sugarcane	8	13	3.2	1.1-9.3
Pesticides in banana	6	8	3.4	1.0-11.8
Pesticides in other crops	4	21	1.4	0.4-4.3
Insecticides in general	4	17	1.4	0.4-4.5
Insecticides in sugarcane	0	0	-	-
Insecticides in banana	1	6	0.7	0.1-7.1
Insecticides in other crops	3	10	2.0	0.5-8.2
Herbicides in general	13	32	2.2	1.0-4.8
Herbicides in sugarcane	8	13	3.2	1.1-9.3
Herbicides in banana	6	7	3.6	1.0-13.2
Herbicides in other crops	4	16	1.9	0.6-6.1
Fungicides in general	2	18	0.8	0.2-3.9
Fungicides in sugarcane	0	0	-	-
Fungicides in banana	1	5	1.9	0.2-18.1
Fungicides in other crops	1	12	0.6	0.1-5.2
Sugarcane work				
Cutting of the sugarcane	7	18	1.7	0.6-4.8
Collecting the sugarcane	5	16	2.3	0.7-7.3
Burning of the sugarcane	4	15	0.7	0.2-2.7
Other work in the field	3	14	1.1	0.2-4.7
Crushing of the sugarcane	2	1	6.1	0.2-157.9
Handling the sugarcane	9	20	2.5	1.0-6.3
